# Therapeutic and prognostic features in myasthenia gravis patients followed in a tertiary neuromuscular diseases center in Turkey

**DOI:** 10.3389/fneur.2023.1176636

**Published:** 2023-08-03

**Authors:** Aylin Yaman, Fatma Kurtuluş Aydın

**Affiliations:** ^1^Neurology Department, Antalya Training and Research Hospital, Antalya, Türkiye; ^2^Ankara Etlik City Hospital, Ankara, Türkiye

**Keywords:** myasthenia gravis, treatment, autoantibodies, thymectomy, COVID-19, Turkey, electromyography

## Abstract

**Introduction:**

In this study, we aim to evaluate the treatment responses and prognostic characteristics of Myasthenia Gravis (MG) patients followed in a tertiary neuromuscular diseases center in Turkey.

**Methods:**

One hundred seventy four MG patients (between years 2011 and 2022) in Antalya, Turkey were diagnosed, and evaluated on a classification of MG was based on Myasthenia. Gravis Foundation of America (MGFA) clinical classification. Exclusion of other possible diseases in the differential diagnosis and support by beneficial response to treatment with acetylcholinesterase inhibitors were also taken into consideration.

**Results:**

Mean age of participants was 54.86 (SD = 14.856; min-max = 22–84). Ninety (51.7%) were female. MG was more common in women under the age of 65 (58%) and in men over the age of 65 (64%). Generalized MG was seen in 75.3% of the patients. Anti-AChR positivities were detected in 52.3%, Anti-MuSK positivity in 4.6%, and seronegativity in 22.4%. Thymoma was detected in nearly 9.8% and thymectomy was performed in 28.7 percent. Most of the patients (57.5%) were using corticosteroids. Azathioprine was used by 39% and mycophenolate mofetil by 10.3% of patients. Mortality was higher and disease was more severe in late-onset (>50 years) MG patients (especially in the COVID-19 pandemic). Eight patients (four women, four men, mean age 75.5 years) died during follow-up. None of them died due to myasthenic worsening, two died due to malignancy and two due to infection. During the COVID pandemic, 16 patients (9.2%) had COVID infection. Four patients died due to COVID-19 infection, these four patients had serious comorbidities, and three of them were elderly (>75 years).

**Conclusion:**

In conclusion, MG is more common in women between the ages of 20–40 and in men over the age of 65. The use of corticosteroids was more common under the age of 50, and the use of non-steroidal immunosuppressant agents was more common over the age of 50. Thymectomy is still an important supportive treatment approach in anti-AChR positive and seronegative generalized patients under 50 years of age. IVIG and plasmapheresis are effective treatments during acute exacerbations and bridging periods of treatments. Specific treatments are needed especially for resistant group of patients.

## Introduction

Autoimmune Myasthenia gravis (MG) is the most common disease affecting the neuromuscular junction in skeletal muscles (1), which is an antibody-mediated disease. The main finding of the disease is fluctuating muscle weakness, especially in ocular, bulbar, respiratory and extremity muscles (2). The number of patients diagnosed with MG is increasing all over the world, and its prevalence is approximately 12.4 per 100,000 (3). MG is more common in women aged 20–40 years, and more common in men over 50 years of age.

Autoantibodies against acetylcholine receptors (AChR) are present in most patients, while autoantibodies against muscle specific kinase (MuSK), low-density lipoprotein receptor-related protein 4 (Lrp4), and agrin can be found less frequently.

MG may be classsified based on clinical manifestations (ocular or generalized), age of onset (before 50 years and after 50 years), antibody status (anti-AChR, anti-MuSK, anti-LRP4, seronegative) and thymus pathology (normal/atrophic thymus pathology, thymic hyperplasia and paraneoplastic occurrence associated with thymoma).

In generalized MG, anti-AChR antibody positivity is 80%–85% and anti-MuSK antibody positivity is around 4%–5% (2).

Presence of thymoma is seen in 10%–15% of cases. It is thought that the patients who are seronegative for both antibodies, who do not have thymoma have a milder clinical picture, and those who are associated with thymoma, who have a late onset (>50 years) and who are anti-MuSK antibody positive have a more severe clinical picture (3). Anti-MuSK positive disease is more frequently seen in young women, and it progresses more severely and more frequently with bulbar involvement (4). It is estimated that up to 60% of ocular-onset cases will evolve to the generalized form within the first 2 years. Anti-AChR antibody is positive in approximately 50% of cases with ocular MG, anti-MuSK antibody positivity is much rarer (3%–4%). Ocular MG patients who are seronegative for both antibodies are much less likely to develop the generalized form than those who are antibody positive. There are studies that have found that late-onset ocular MG cases are more likely to be generalized (5).

MG has turned historically from a disease with a poor prognosis to a well-managed and treatable disease in recent years, with the widespread use of immunosuppressive and immunomodulatory therapies, the use of thymectomy in appropriate patients, and close patient follow-up. However, there are cases that are resistant to treatment, albeit at a low rate. By the 2000s, the mortality of the disease has decreased significantly (3%–4%).

Respiratory failure seen with myasthenic crisis is the leading cause of death (6, 7).

In this study, we aimed to reveal the treatment responses and prognostic characteristics of MG patients followed in a tertiary neuromuscular diseases center in Turkey.

## Materials and methods

This cohort includes all the autoimmune MG patients who had been followed-up in tertiary neuromuscular diseases center in Ministry of Health Antalya Education and Research Hospital between the years 2011 and 2022 in Antalya, Turkey.

One hundred seventy four patients were included to the study. Diagnosis was based on the muscle weakness or fatigue confirmed in examination, antibody testing, electrophysiological tests consistent with neuromuscular transmission impairment, chest images to detect the thymic pathologies.

EMG tests included repetitive nerve stimulations and jitter measurements.

Exclusion of other possible diseases in the differential diagnosis and support by beneficial response to treatment with acetylcholinesterase inhibitors were also taken into consideration.

Myasthenia Gravis Foundation of America (MGFA) clinical classification was used for the documentation of clinical severity at the time of application. MGFA-Post Intervention Status (PIS) was used as an outcome measure (8).

The adverse effects of corticosteroids (CS), which are the most commonly used agents in the treatment of MG, were defined as “mild” and “serious”. Mild adverse effects were accepted as effects like mild weight gain and glucose intolerance; severe adverse effects were accepted as severe weight gain, cushingoid appearance, vertebral fracture, cataract formation, glaucoma, femoral head avascular necrosis, osteoporosis, overt diabetes, neuropsychiatric disturbances etc. In maintenance use, low dose was accepted as below 20 mg/day for methyl-prednisolone and below 25 mg/day for prednisolone.

Refractory MG is defined as ‘PIS is unchanged or worse after CS and at least 2 other immunosuppressive agents, used in adequate doses for an adequate duration, with persistent symptoms or side effects that limit functioning, as defined by patient and physician’ (9).

Ethics committee approval was obtained. It was obtained from the ethics committee of Antalya Training and Research Hospital.

Statistical analysis was performed as follows: non-parametric tests were used for data showing ordinal and quantitative distribution, and parametric tests were used for data showing continuous distribution. Alpha was accepted as 0.05 as the significance level.

## Results

The mean age of participants was 54.86 (SD = 14.856; min-max = 22–84; *n* = 174). Ninety (51.7%) were female and 84 (48.3%) were male. Ninety nine (56.9%) of the patients were above 50 and 43 (24.7%) of them were above 65 years of age. MG was more common in women under 65 years of age (*n* = 76; 58%) and in men over 65 years of age (*n* = 29; 64%) (*p* < 0.05). The mean duration of their diseases was 7.21 (SD = 7.055; min-max = 1–37) years. The mean follow-up duration of patients was 4.84 (SD = 2.748; min-max = 1–11) years, median follow-up was 5 years. Patients under 65 years of age had longer duration of illness (mean = 7.93; SD = 7.604; *n* = 131 vs. mean = 5.02: SD 4.421; *n* = 43) (*p* < 0.05). Sole ocular involvement was evident in 43 (24.7%) and generalized involvement in 131 (75.3%) patients. Anti-AChR positivity was detected in 91 (52.3%), Anti-MuSK positivity in 8 (4.6%), seronegativity in 39 (22.4%) patients. Since the anti-MuSK antibody level cannot be measured in the hospital laboratory and examination outside the hospital is not covered by the general insurance, 35 (20%) of patients were found to be anti-AChR antibody negative and anti-MuSK antibody status of these patients was not known. These patients were excluded from the analysis when determining the disease characteristics and outcome of anti-AChR positive vs. anti-MuSK positive vs. seronegative patients. Anti-AChR antibody (*n* = 58; 64%) was more common at the age of 50 and over, and anti-MuSK positivity was more common in women (*n* = 7; 88%) under the age of 50 (*n* = 7; 88%). Anti-titin antibody was positive in one patient (Anti-titin antibody was not investigated in the rest of the patients). No paraneoplastic etiology was found in this patient. In generalized form, anti-AChR antibody (*n* = 73; 57% vs. *n* = 18; 42%) was higher than anti-MuSK (*n* = 8; 6% vs.0) and seronegativity (*n* = 11; 26% vs. *n* = 28; 22%) was found to be lower (*p* < 0.05), when compared to ocular form. Under 50 years of age (clinical ocular, *n* = 16; 21%; clinical generalized, *n* = 59; 79%) and above (clinical ocular, *n* = 27; 28%; clinical generalized, *n* = 72; 72%) in terms of clinical features, there was no difference between groups (*p* > 0.05). Seventeen (9.8%) patients had thymoma. Thymectomy was performed in 50 (28.7%) patients. Pathological evaluation revealed benign results in 37 (21.3%), malign thymoma in 10 (5.7%) patients, and pathology records could not be reached in 3 (1.7%) patients (they declared the result as benign). There was no difference between the age of 50 and older in terms of the presence of thymoma (*p* > 0.05). Thymic hyperplasia (*n* = 11; 48%) was more common in patients under 50 years of age (*p* < 0.05). Thymectomy (*n* = 37; 76%) was performed more frequently in patients under 50 years of age (*p* < 0.05).

The summary of patients’ demographic and disease characteristics are shown in Table 1.

**Table 1:** Patients’ demographic and disease characteristics.

Category	
	**Mean (SD; min-max)**
Age	54.9 (14.8; 22–84)
Mean duration of disease	7.2 (7.0; 1–37)
Mean follow-up duration	4.8 (2.7; 1–11)
	**Number (percentage)**
Female	90 (52%)
Male	84 (48%)
≥ 50 years Female	40 (44%)
<50 years Female	50 (56%)
≥ 50 years Male	61 (73%)
<50 years Male	23 (27%)
Ocular MG	43 (25%)
Generalized MG	131 (75%)
Anti-AChR +	91 (52%)
Anti-MuSK +	8 (5%)
Seronegative	39 (22%)
Patients underwent thymectomy	50 (29%)
Patients under the age of 50 who had thymectomy	37 (76%)
Patients with thymoma	17 (10%)
MGFA at the time of application	
I	46 (26%)
II	14 (8%)
IIA	26 (15%)
IIB	41 (23%)
IIIA	12 (7%)
IIIB	22 (13%)
IVA	2 (1%)
IVB	11 (6%)
MGFA-PIS at the last visit	
Complete stable remission	1 (1%)
MM-0	13 (7%)
MM-1	35 (20%)
MM-2	56 (32%)
MM-3	51 (29%)
Change in status, improved	10 (6%)
Change in status, unchanged	8 (5%)

MGFA, Myasthenia Gravis Foundation of America (Clinical classification of MG); MGFA-PIS, MGFA Post-Intervention Status, MM, Minimal Manifestations.

Thymus pathologies were found to be more benign (*n* = 31; 84%) in patients under 50 years of age (*p* < 0.05). Most of the patients used CS (*n* = 101; 57.5%) alone or in combination with other immunosuppressants. Short-term (<6 months) CS use was present in 36 (20.7%) and long-term (>6 months) use in 65 (37.4%) patients. Most chronic CS users (*n* = 98; 56.3%) used low dose and remaining (*n* = 3; 1.7%) used high dose. Mild adverse effects were reported in 36 (20.7%), severe in 8 (4.6%) of the patients.

Azathioprine (AZA) was used by 68 (39%) patients. Mycophenolate mofetil (MM) was used by a total of 18 (10.3%) patients; in 17 (25% of AZA users) of these patients, AZA was prescribed as first-line steroid sparing immunosuppressive agent, but they switched from AZA to MM because of adverse effects. Mostly these adverse effects were the persistent elevation of liver enzymes more than twice the normal levels or neutropenia, which normalized after discontinuation of the drug. Only in one (1.5% 0f AZA users) of the patients, a 67 years old female, severe AZA-induced neutropenia developed lasting about 2 weeks, she was hospitalized and followed-up with hematology clinic, and finally her values returned to normal. Two months later, MM was prescribed, she is under MM treatment with good response and no adverse effects for 5 years. In the remainder of the patients who developed AZA-related adverse events, the effects were transient and resolved with drug discontinuation.

There were no remarkable side effects in patients using MM.

Intravenous immunoglobulin (IVIG) was used in 96 (55.2%) patients. Most had a shorter term (<6 months), 22 (12.6%) longer term (≥ 6 months) IVIG use, and 4 (2.3%) patients who are refractory to single or combination immunosuppressant treatment or with unacceptable side effects, need IVIG as a maintenance treatment with other immunosuppressive agents. Three of these refractory patients are currently under low dose oral CS, MM and IVIG treatment. One of the refractory patients has also rheumatoid arthritis diagnosis, using another immunosuppressive agent, CS and IVIG. Two of these refractory patients were anti-AChR positive and two of them were seronegative, three of them were above 50 years, one was below 50 years. All four of them continue their lives with mild to moderate symptoms.

Only in one (1% of IVIG users) patient, a potentially serious side effect, unstable angina pectoris and temporary elevation of troponin was observed due to IVIG.

Patients under 65 years of age had more CS (*n* = 46; 63% vs. *n* = 18; 42%), less AZA (*n* = 40; 31% vs. *n* = 30; 70%) and less IVIG (*n* = 63; 50% vs. *n* = 33; 77%) (*p* < 0.05). AZA use increased with increasing age (*n* = 70; mean = 59.09; SD = 17.709 vs. *n* = 102; mean = 47.12; SD = 16.486) (*p* < 0.05). AZA use (*n* = 51; 52% vs. *n* = 19; 26%) was more common in patients over 50 years of age (*p* < 0.05).

CS use (*n* = 57; 76% vs. *n* = 43; 44%) was found to be more common under the age of 50 (*p* < 0.05).

Plasmapheresis was used in 11 (6.3%) patients during exacerbations.

There was no difference in terms of hospitalization in the intensive care unit between the groups under 50 years old and over (*p* > 0.05). In terms of myasthenic crisis, there was no difference between the groups under 50 years old and over (*p* > 0.05).

Seven (4%) patients used Rituximab (RTX), four of them were anti-MuSK and three were anti-AChR positive patients. Six of these patients had a favorable response to RTX, one anti-AChR positive patient did not respond well. No significant adverse effect was observed due to RTX.

Remaining four of the anti-MuSK positive patients are clinically stable under quite low dose of CS, and did not need another immunosuppressant agent.

During the follow-up, eight patients (4 females, 4 males, mean age 75.5) died. None of them died because of myasthenic worsening. Two of the patients died due to malignancy and two due to non-COVID infection. Four patients died due to COVID-19 infection; these four patients had serious co-morbidities, and three of them were elderly (>75 years).

One hundred fifty six (89%) patients were in complete stable remission or, mostly, minimal manifestations state according to MGFA-PIS classification (detailed clinical status information is given in Table 1). There was no difference in clinical status between patients younger than 50 years of age and older (*p* > 0.05).

The distribution of treatments is shown in Table 2.

**Table 2:** Number of patients under different treatments and their clinical and serologic features.

	AZA	MM	RTX	Only CS	CS + MM + IVIG	Only symptomatic (pyridostigmine)
	*n*:51	*n*:18	*n*:7	*n*: 50	*n*:4	
	(29.3%)	(10.3%)	(4%)	(28.7%)	(2.3%)	
						*n*:44 (25.2%)
Generalized	51	18	7	36	4	3
Ocular	–	–	–	14	–	41
AchR Ab +	43	15	3	22	2	6
MuSK Ab +	–	–	4	4	–	–
Seronegative	8	3	–	14	2	12

AZA, Azathioprine; MM, Mycophenolate mofetil; RTX, Rituximab; CS, Corticosteroids; IVIG, Intravenous immunoglobulin.

## Discussion

According to our findings, MG was more common in women under 65 years of age (*n* = 76; 58%) and in men over 65 years of age (*n* = 29; 64%) (*p* < 0.05). This is compatible with the literature, supporting the bimodal distribution of the disease (2, 10, 11). In the study conducted by Mercelis et al. including a single center in Belgium, women were slightly more than men (53%) (12). In another study (1,060 patients with MG and covering the years 1980–2008), it was found that, the disease started in 66% of men and 42% of women at the age of >50 years (13). Grob et al. reported that MG can occur at any age, but it is more common in women (14). Findings of all these studies show similarities to the gender distribution of our patients. There are some explanations why it is more common in women at an early age. It is thought that sex hormones may particularly affect the production of antibodies (15, 16). Additional findings related to female patients in our study were that, apart from being younger, they had MG for a longer period of time and they needed thymectomy more frequently.

Frequencies of generalized and ocular forms of disease in our patient group showed no significant difference between early and late onset subgroups (>50 and < 50 years of age).

In our study, Anti-AChR antibody positivities were detected in 52.3% of the patients, Anti-MuSK antibody positivity in 4.6%, and seronegativity in 22.4%. Anti-AChR antibody (*n* = 58; 64%) positivity was more common at ≥ 50 years of age, while anti-MuSK positivity was more frequent at <50 (88%) years and in women (88%).

The prevalence of anti-MuSK positive MG varies from country to country. It is low in Northern Europe and rises towards the Mediterranean. In Japan, the prevalence of anti-MuSK antibodies was reported to be 2–3%. Four of the total 8 Anti-MuSK positive patients (100% women <50 years old and 50% of all Anti MuSK positive cases) in our group, showed good clinical course with low dose steroid, in these cases no interventions such as non-steroidal immunosuppressant use or long-term IVIG use were required. Although our patient group is small in number, it is not consistent with the general impression that anti-MuSK positive cases have more severe clinical course (5).

In a study conducted in 13 European countries, the prevalence of seronegative MG cases was found to be 5%–22% (17–22).

The presence of thymoma makes MG a paraneoplastic disease and is present in 10%–15% of MG patients (23). In our patient group, thymic hyperplasia and benign thymus pathologies were found more frequently in the younger patients. Seventeen (9.8%) patients had thymoma. Thymectomy was performed in 28.7% patients.

Pathological evaluation revealed benign results in 21.3%, malign thymoma in 5.7% of all thymectomized patients. Cases with thymoma and especially malignant thymoma are expected to have a more severe clinical course, which is the case in our patient group as well.

Patients with lymphoid follicular hyperplasia or normal thymus have a better prognosis. They are a possible source of antibodies that develop hyperplastic thymus, especially in AChR Ab positive MG patients. This is confirmed by clinical improvement (up to 85%) after thymectomy. In the International thymectomy trial (MGTX), in MG patients without thymoma, better clinical outcomes were achieved at three-year follow-up after thymectomy. When the 5-year results after thymectomy were evaluated, 61% of all patients were followed up, and it was understood that the treatment of patients with thymectomy was more successful, they used less CS and required less hospitalization (24). Based on the success of this study, thymectomy was performed more frequently in early-onset generalized MG patients with AChR antibodies. Minimally invasive thymectomy surgery has facilitated this process (25, 26). Thymectomy is not recommended in MuSK Ab positive patients (23).

Consistent with this evidence, thymectomy (*n* = 37; 76%) was performed more frequently in patients under 50 years of age (*p* < 0.05) in our patient group. Thymus pathologies were found to be more benign (*n* = 31; 84%) in patients under 50 years of age (*p* < 0.05).

Most of our patients used steroids (57.5%). Short-term (< 6 months) steroid use was present in 36 (20.7%) and long-term (> 6 months) use in 65 (37.4%) patients. Most steroid users (56.3%) used low dose and remaining (1.7%) used high dose steroids. Mild adverse effects were reported in 20.7%, severe in 4.6% of the patients.

Azathioprine (AZA) was used by 68 (39%) of our patients. It was the most frequently used steroid-sparing immunosuppressive agent in our patient group. In 18 (25%) patients AZA was prescribed as first-line immunosuppressive agent, but it was discontinued because of adverse effects. Mostly these adverse effects were the elevation of liver enzymes more than twice the normal levels or neutropenia, which normalized after discontinuation of the drug. Only in one of the patients (1.5% of AZA users) a severe side effect (neutropenia) was observed, which was reversible. In general, AZA, as a classical immunosuppressant in the treatment of generalized MG, is a highly effective and easily tolerated drug that limits the use of steroids, although the rate of discontinuation due to the development of side effects at the beginning of the treatment can be considered high (25%). Our general observation is that after 3–4 years of AZA use, the disease goes into remission and dose reductions can be made.

Eighteen (10%) of our patients used mycophenolate mofetil (MM) and the need for drug discontinuation was not observed due to side effects. Its side effects are rare. It can be interpreted that it is a well tolerated drug. Our three of the 4 refractory patients who needed maintenance IVIG treatment were using CS and MM. This suggests that MM in MG may be a less effective drug than AZA, although the number of patients is not too large to make a general comment. In the study of Hehir et al., in which the outcomes in 102 patients were documented, it was found to be effective in 50% of patients who received MM, and in the second year, this success was 80% (27). It is recommended for mild and moderate MG, and it is generally used in addition to CS when the effect is low (28).

RTX was used in 4% of patients, four (57%) anti-MuSK and three (43%) anti-AChR positive. Six of them (85%) responded well to RTX, whereas one patient (15%) positive for anti-AChR did not respond to treatment. No significant side effects were observed due to RTX.

Besides its use in myasthenic crisis and exacerbations, four (2.3%) of our patients who are refractory to treatment, needed IVIG as a maintenance treatment with other immunosuppressive agents. Two of these refractory patients were anti-AChR positive and two of them were seronegative. In cases where prednisone combined with another immunosupressant is not sufficient for symptomatic improvement, a single or multiple doses of IVIG are given in cases of bulbar involvement. In chronic use, it is well tolerated and reduces the need for immunosuppressants and acetylcholine esterase inhibitors (29, 30).

Only one (1% of IVIG users) of our patients experienced a serious side effect (elevation of troponin and angina pectoris), but it could be managed.

Two anti-AChR positive and resistant patients who are under adequate immunosuppressant medication but still in need of maintenance IVIG treatment are candidates for second-line treatment (e.g., Eculizumab) (6).

It has been reported that the treatment of late-onset MG patients is more complex and the comorbidity is higher. In their study Cortés-Vicente et al. revealed that late-onset MG patients were mostly male, had more anti-AChR antibodies, had more ocular involvement (31). In a study originating from Turkey, it was stated that the prognosis of late-onset MG was better (32). However, the outputs of our study is contradictory to this finding, since our patients older than 65 years used AZA (*n* = 30, 70% vs. *n* = 40, 31%) and IVIG (*n* = 33, 77% vs. *n* = 63, 50%) more frequently (*p* < 0.05), suggesting that they had more severe course.

As age increases, comorbidity also increases, in this context, mortality was higher and clinical course was more severe in late-onset (>50 years) MG patients (especially in the COVID-19 pandemic), and this affects our treatment choice and success of treatment (especially MG patients after >65 years of age needed more frequent IVIG therapy).

One hundred fifty six (89%) patients were in complete stable remission or, mostly, minimal manifestations state according to MGFA-PIS classification.

Eight patients (four women, four men, mean age 75.5 years) (5%) died during follow-up. None of them died due to myasthenic worsening, two died due to malignancy and two due to non-COVID, four due to COVID-19 infection. A Swedish database study including 4,559 MG patients, concluded that, death rate in patients with MG was not different from the death rate of the Swedish population. The most common cause of death was cancer (19.5%). Deaths in MG patients (11%) were 2.5 times more common due to influenza or pneumonia (33). The presence of thymoma indicates a poor prognosis (34).

During the COVID-19 pandemic, 16 patients (9.2%) had COVID-19 infection. Overall, 75% of these patients did not progress more severely than the general population, and all patients continued their immunosuppressant treatment. Four patients died due to COVID-19 infection, these four patients had serious comorbidities, and three of them were elderly (>75 years). One of the patients who died of COVID-19, 56 years of old male, had a malign thymoma history. MG patients were more adversely affected by the current immune suppression, weakness of respiratory muscles and respiratory failure, pneumonia and pulmonary thromboembolism during the COVID-19 pandemic. There is also an increase in the mortality rate due to ARDS, which occurs with immune dysregulation and respiratory muscle insufficiency (35). When the frequency of COVID-19 was examined in a French MG cohort (CO-MY-COVID registry), COVID-19 was detected in 0.96% (*n* = 34) of 3,558 MG patients. Five patients died. The immunosuppressants and CS they used did not adversely affect the result (36), just like in our patient group. In a retrospective study conducted in Brazil, the care processes of 15 MG patients with COVID-19 were examined. It was concluded that immunosuppressant treatments had no adverse effects on the clinical course and should be continued in patients with COVID-19 (37). These studies are also consistent with our experiences during the COVID-19 pandemic. In a systematic review, it was reported that among the risk factors that increase the severity of the disease in MG patients with COVID-19, severe MG clinic, old age, long-term steroid use, use of RTX, and comorbidities (35).

The limitations of this study are that it is single-centered and descriptive. However, the scarcity of studies on this subject, the transfer of clinical experience, and the reflection of experiences during the COVID-19 pandemic are among the important outputs of this study.

In conclusion, the age and gender distributions of the patients in our study group are similar to previous studies. MG is more common in women between the ages of 20–40 and in men over the age of 65. The use of corticosteroids was more common under the age of 50, and the use of non-steroidal immunosuppressant agents was more common over the age of 50. AZA is a clinically effective and safe agent that should be used with close follow-up as a classical and first-choice steroid-sparing immunosuppressant agent. MM is seen as an agent with no serious side effects, but whose efficacy may remain low in severe cases. RTX, on the other hand, seems to be effective and safe, especially in anti-MuSK positive cases. Thymectomy is still an important supportive treatment approach in anti-AChR positive and seronegative generalized patients under 50 years of age. IVIG and plasmapheresis are effective treatments during acute exacerbations and bridging periods of treatments. MG is a disease that can be managed very well with close and individualized follow-up. There is a small proportion of resistant patients who do not respond to conventional treatments. In these patients, complement inhibitors can be tried in those who are anti-AChR antibody positive. These agents also have uncertainities such as duration of treatment, long-term side effects, and cost issues. Specific treatments are needed especially for this group of patients.

## Data availability statement

The data analyzed in this study is subject to the following licenses/restrictions: these are patient records of the hospital. Requests to access these datasets should be directed to antalyaeah@saglik.gov.tr.

## Ethics statement

The studies involving human participants were reviewed and approved by Antalya Training and Research Hospital, Ethical Committee. Written informed consent for participation was not required for this study in accordance with the national legislation and the institutional requirements.

## Author contributions

All authors listed have made a substantial, direct, and intellectual contribution to the work and approved it for publication.

## Conflict of interest

The authors declare that the research was conducted in the absence of any commercial or financial relationships that could be construed as a potential conflict of interest.

## Publisher’s note

All claims expressed in this article are solely those of the authors and do not necessarily represent those of their affiliated organizations, or those of the publisher, the editors and the reviewers. Any product that may be evaluated in this article, or claim that may be made by its manufacturer, is not guaranteed or endorsed by the publisher.
